# Management of anticoagulation and antiplatelet therapy in patients with primary membranous nephropathy

**DOI:** 10.1186/s12882-019-1637-y

**Published:** 2019-12-02

**Authors:** Honghong Zou, Yebei Li, Gaosi Xu

**Affiliations:** Department of Nephrology, the Second Affiliated Hospital of Nanchang University, 330006; No. 1, Minde Road, Donghu District,, Nanchang, People’s Republic of China

**Keywords:** Idiopathic membranous nephropathy, Thromboembolic complications, Anticoagulation, Review

## Abstract

**Background:**

It has been recognized that primary membranous nephropathy (MN) is related to an increased risk for thromboembolic complications. However, the current evidence supporting prophylactic and therapeutic anticoagulation is too weak to better meet the clinical needs of this patient population. The present review provides some suggestions to guide the decision on anticoagulant management in primary MN patients with a high risk of thrombosis or with thromboembolic complication.

**Materials and methods:**

We extracted relevant studies by searching the published literature using the Cochrane Library, Medline, PubMed and Web of Science from March 1968 to March 2018. Eligible publications included guidelines, reviews, case reports, and clinical trial studies that concerned the rational management of anticoagulation therapy in the primary MN population. The evidence was thematically synthesized to contextualize implementation issues.

**Results:**

It was helpful for clinicians to make a decision for personalized prophylactic aspirin or warfarin in primary MN patients when serum albumin was < 3.2 g/dl to prevent arterial and venous thromboembolic events (VTEs). The treatment regimen for thromboembolic complications (VTEs, acute coronary syndrome and ischemic stroke) in primary MN was almost similar to that for the general population with thromboembolic events. It is noteworthy that patients should continue the previous primary MN treatment protocol during the entire treatment period until they achieve remission, the protocol is complete and the underlying diseases resolve.

**Conclusion:**

The utility of prophylactic aspirin or warfarin may have clinical benefits for the primary prevention of thromboembolic events in primary MN with hypoalbuminemia. It is necessary to perform large randomized controlled trials and to formulate relevant guidelines to support the present review.

## Background

Primary membranous nephropathy (MN) is one of the leading causes of nephrotic syndrome (NS) in adults [1]. The aims of therapy in primary MN have mainly focused on the prevention of end-stage renal disease (ESRD), which usually occurs after several years, whereas other complications of primary MN may occur much earlier in the course of the disease [2]. Venous thromboembolic events (VTEs), including deep venous thrombosis (DVT), renal vein thrombosis (RVT) and pulmonary embolism (PE), are recognized as early complications of primary MN that carry significant morbidity and mortality [3]. In particular, hypoalbuminemia is the most significant independent indicator of VTE risk [4]. Along with VTEs, high absolute risks of arterial thromboembolic events (ATEs) were remarkably elevated within the first 6 months after presentation. Severe proteinuria, estimated glomerular filtration rate and smoking were predictors of ATEs [5–7]. The primary cardiovascular events (CVEs) included acute coronary syndrome (ACS) and ischemic stroke (IS). It was reported in a Chinese study that 36% of primary MN patients had a VTE, 33% had an RVT and 17% had a PE [8]. Recent research has provided novel data on the incidence of CVEs in primary MN [9]. The morbidity of CVEs was 4.4, 5.4, 8.2, and 8.8% at 1, 2, 3, and 5 years, respectively, in a primary MN cohort. In addition, they found that the incidence of CVEs was at least as high as that of ESRD early in the course of the disease. Therefore, a reduction in CVEs should be considered as a focus of intervention and as a therapeutic outcome measure in primary MN.

Thus, it is extremely important to implement measures to prevent thromboembolic events as part of the daily support care for primary MN patients with hypoalbuminemia. Unfortunately, the current evidence of the 2012 KDIGO supporting prophylactic and therapeutic anticoagulation is too weak to better meet the clinical needs of this patient population, given the need to carefully manage anticoagulants and antiplatelet agents, and to tailor the therapeutic regime to an individual’s risks of thromboembolic events and hemorrhage, we have conducted the present review to provide some suggestions to guide the decision on anticoagulant management in primary MN with a high risk of thrombosis or with thromboembolic complications.

## Methods

We extracted relevant studies by searching the published literature using the Cochrane Library, Medline, PubMed and Web of Science from March 1968 to March 2018. The PRISMA Extension Checklist for Scoping Reviews (PRISMA-ScR) was attached to make sure the present review have included all important standard elements (PRISMA-ScR-Checklist in Supplementary Material). The publications with no title or no abstract and those not in English were excluded. The overall results were analyzed after the first screening to present an overview of the existing literature in management of anticoagulation in patients with primary MN. Subsequently, we focused on the literature presenting original research to identify the use of prophylactic and therapeutic anticoagulation in primary MN patients with high risks of thrombosis or with thromboembolic complications in the existing research. However, the studies surrounding the use of prophylactic and therapeutic anticoagulation in patients with primary MN were relatively limited. We expanded the scope of the search and studies were included regardless of small sample size. In addition, references from relevant articles were examined for additional content not found during the initial search.

The predefined key search terms included MN, membranous glomerulonephritis, NS, prophylactic anticoagulation, anticoagulation, antiplatelet therapy, warfarin, heparin, unfractionated heparin (UFH), low molecular weight heparin (LMWH) and direct oral anticoagulant (DOAC), thromboembolic complication, thromboembolic disease, thrombosis, thromboembolism, thrombotic events, cardiovascular events, venous thromboembolic event, deep venous thrombosis, renal vein thrombosis, pulmonary embolism, arterial thromboembolic event, acute coronary syndrome, and ischemic stroke.

## Pathogenesis of thrombogenesis in primary MN

At present, the pathogenesis of thrombogenesis in NS is not absolutely clear, but it seems to be multifactorial. Several mechanisms that promote thrombosis in NS have been identified. First, an alteration in plasma levels of fibrinolysis and coagulation, along with the urinary loss of proteins, can lead to lower levels of proteins such as antithrombin, protein C and protein S [10, 11]. The disease-specific risk of VTE is dependent on the levels of proteinuria and serum albumin, as well as cancer history. Second, the risk of an ATE is attributed to increased platelet activation and aggregation [12]. There are of course other factors that can predispose to thromboembolism such as hyperviscosity, hyperlipidemia, previous thromboembolic episodes, and steroid therapy itself, which is commonly used in the treatment of NS; steroid therapy can cause hypercoagulability and provoke thrombosis [13].

## Results: prevention of thrombogenesis and the anticoagulant management of primary MN

The risk of thromboembolic events is particularly high in primary MN when compared with other pathological types of NS [14], and most of the patients remain asymptomatic [8]. Therefore, it seems attractive to consider the prophylactic use of anticoagulants or antiplatelet agents to prevent VTEs and ATEs in primary MN patients with high thromboembolic risk. Moreover, the rational management of therapeutic anticoagulation and antiplatelet agents in primary MN patients with thromboembolic complications may reduce the recurrence risk of CVEs.

### Prophylactic anticoagulation for primary MN

Evidence has shown that low serum albumin is a strong independent risk factor for VTEs in primary MN [4, 15]. A retrospective study indicated that the increasing risk was proportionately associated with declining albumin levels. Each 1.0-g/dl decrease in albumin level was associated with a 2.13-fold increased risk of VTE. The threshold albumin level identified for the overall risk of VTEs was 2.8 g/dl. In other words, a serum albumin level < 2.8 g/dl meant a high risk of a VTE [4]. In view of the high risk of thromboembolic complications in primary MN, anticoagulation was warranted in patients who initially presented with thrombotic events [16]. However, controversy exists about the use of prophylactic anticoagulation therapy in primary MN.

The 2012 KDIGO suggests that prophylactic oral warfarin can be considered in primary MN patients once serum albumin is < 2.5 g/dl and there are additional risks for thrombosis, whereas some physicians think that prophylactic anticoagulation should be initiated earlier. Evidence suggests that aspirin has a therapeutic benefit for the primary prevention of VTEs and the recurrence of VTEs and results in a significant reduction in the rate of major vascular events with no apparent increase in the risk of major bleeding [17, 18]. As the pathogenesis of NS was associated with platelet hyperactivity, some investigators advocated that primary MN patients could be administered antiplatelet agents such as aspirin for the primary prevention of thrombotic events at an early stage of disease [19, 20].

The benefits of anticoagulation in preventing VTEs must be weighed against the risk of hemorrhage complications in individual patients. Lee et al. constructed a Markov-based decision analysis model to estimate the possibility of benefit based on an individual’s bleeding risk profile, serum albumin level, and acceptable benefit-to-risk ratio (http://www.med.unc.edu/gntools/) [21]. They estimated that 4.5 VTEs would be prevented at the cost of one major bleed during 2 years of therapy with prophylactic anticoagulation (benefit-to-risk ratio = 4.5:1) in patients with a low bleeding risk [Anticoagulation and Risk Factors in Atrial Fibrillation risk (ATRIA) score 0 ~ 3 out of 10] and serum albumin < 3.0 g/dl [22]. However, these estimates were based on a retrospective cohort study with relatively small sample sizes. Therefore, it may not be possible to assess the generalizability/external validity of these estimates.

Owing to the 0.1% risk of a major bleeding complication with aspirin, the risk is deemed too high to balance the benefit of a 25% risk reduction in the general population with a baseline ATE risk of 5 per 1000 patient-years (py) [23]. In other words, a benefit-risk-ratio of 4:1 represents an absolute ATE risk of 20 per 1000 py in a population [20]. If we accept a benefit-risk-ratio of 4:1, prophylactic therapy seems acceptable in this population. Hofstra et al. suggested that primary MN patients might not need to receive prophylactic anticoagulation when serum albumin is > 3.2 g/dl [20]. For patients with a serum albumin of 2.5 ~ 3.2 g/dl, after the evaluation of the ATE risk (https://www.cvdriskchecksecure.com/FraminghamRiskScore.aspx), the researchers did not favor prophylactic anticoagulation if the ATE risk was < 20/1000 py. Prophylactic aspirin therapy seemed acceptable if the ATE risk was ≥20/1000 py. They believed that the utility of aspirin had clinical benefits for primary prevention of ATEs in this patient population [20, 21].

**Fig. 1:**
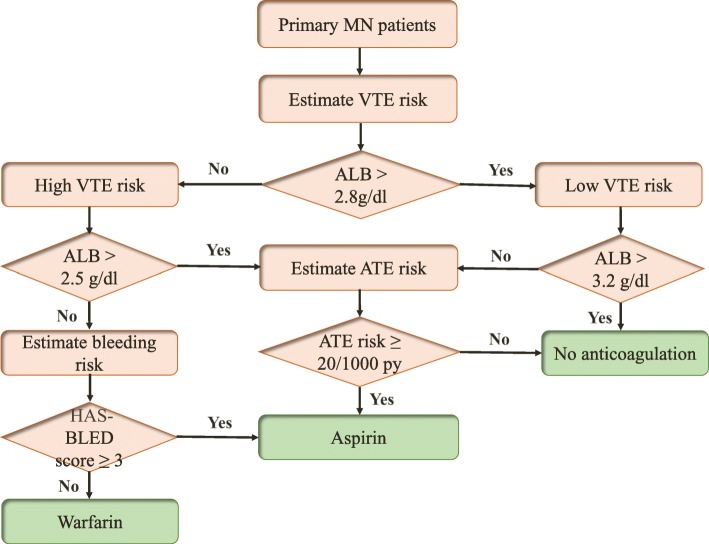
Decision approach for the primary prevention of VTEs and ATEs in primary MN patients. MN: membranous nephropathy; VTE: venous thromboembolic event; ALB: serum albumin; ATE: arterial thromboembolic event; HAS-BLED: hypertension, Abnormal renal/liver function, Stroke, Bleeding history or predisposition, Labile international normalized ratio, Elderly, Drugs/alcohol; HAS-BLED score ≥ 3: high bleeding risk; Adapted from Hofstra et al. [20] and Lee et al. [21]

As the HAS-BLED (hypertension, abnormal renal/liver function, stroke, bleeding history or predisposition, labile international normalized ratio, elderly, drugs/alcohol) bleeding risk score is more predictive of bleeding (cerebral) than the ATRIA score [24], in our own opinion, once serum albumin is < 2.5 g/dl, prophylactic warfarin can be considered in primary MN patients with a HAS-BLED risk score < 3 [25, 26]. In contrast, we suggest that prophylactic aspirin can be given to patients with high bleeding risk (HAS-BLED risk score ≥ 3, 20, 21]. A therapeutic target of the international normalized ratio (INR) is in the range of 2.0 ~ 3.0. Figure 1 guided the decision on the primary prevention of VTEs and ATEs in primary MN patients.

In view of the facts that aspirin does not appear to increase the risk of major bleeding and the pathogenesis of NS is associated with platelet hyperactivity, we are more inclined to implement aspirin as a primary prophylactic anticoagulant for MN patients with a risk of ATEs or VTEs at an early stage of disease. The time of ending anticoagulant therapy should be determined according to relevant indicators such as the levels of proteinuria and serum albumin and the INR. Notably, given that proteinuria may reduce and the level of serum albumin may rise in patients with the treatment of primary MN during the course of disease, the need for any anticoagulation requires constant monitoring. Provided that there is a strong indication that requires anticoagulant therapy in addition to that for primary MN, the patient might have lifelong anticoagulation therapy.

### Primary MN with complications of VTEs

VTEs include DVT and PE, which are the same kind of diseases with two different clinical manifestations and at two different stages. Once the diagnosis of acute DVT (provoked and unprovoked by VTEs) is established 3 months of anticoagulation therapy are the best option for patients, especially in proximal DVT patients, patients with a high recurrence risk of isolated distal DVT, as well as patients with DVT and PE [27, 28].

In patients with DVT not provoked by surgery or cancer, DVT treatment is generally composed of three phases. In the initial period of treatment (5 ~ 21 days following diagnosis), guidelines suggest that patients receive either parenteral therapy for 5 ~ 10 days, such as UFH, LMWH or fondaprinux, and bridge with vitamin K antagonists (VKAs) or use high-dose DOACs. The heparin can be stopped until the INR is maintained in the range of 2.0 ~ 3.0 for 2 days. Then patients are treated with VKAs or DOACs in a period of long-term treatment (first 3 ~ 6 months) [28]. Full or lower doses of LMWH treatment for 4 ~ 6 weeks or ultrasound surveillance could be effective and safe in patients with a low recurrence risk of isolated distal DVT, rather than 3 months of anticoagulation therapy [27–30].

The decision to extend anticoagulant treatment (beyond the first 3 ~ 6 months) is dependent on the patient and physician’s “trade-off” acceptability of bleeding risk and recurrence risk [27, 28, 30]. For patients with multiple VTE episodes, a strong VTE familial history, or a high risk of recurrence, once anticoagulation is stopped, the risk of VTE recurrence in the years after a first episode is consistently approximately 30% [31, 32]. Hence, continuing indefinite anticoagulation with the same drug being administered is the best option in this patient population during the extended treatment. It is also suitable for patients with a first-episode unprovoked VTE and with severe presentation but with low to intermediate bleeding risk to continue indefinite anticoagulation [28, 33]. It is not recommended that patients with high bleeding risk received extended treatment. In patients with unprovoked proximal DVT and a low risk of recurrence, provided that veins are recanalized or stable for 1 year, the discontinuation of anticoagulation can be considered and has proven to be safe in patients who have been repeatedly D-dimer-negative [27, 34]. However, although continuous D-dimer measurement could be used as an indicator to determine whether it is safe to stop anticoagulant therapy in the general population with VTE, a cross-sectional study suggested that D-dimer levels might be increased in association with the degree of proteinuria in NS in the absence of clinically evident DVT [35]. Therefore, D-dimer levels may not be an independent predictor of the cessation of anticoagulant therapy in primary MN with VTE.

Patients with newly diagnosed proximal DVT who are not candidates for anticoagulation could have a removable vena cava filter placed (if there are no contraindications) [36]. For some patients at high risk of PE with or without DVT, reperfusion treatment can be considered if patients have hypotension. UFH should not be stopped in reteplase therapy. However, it is usually recommended that thrombolytic therapy should not be administered in patients without hypotension [27].

**Fig. 2:**
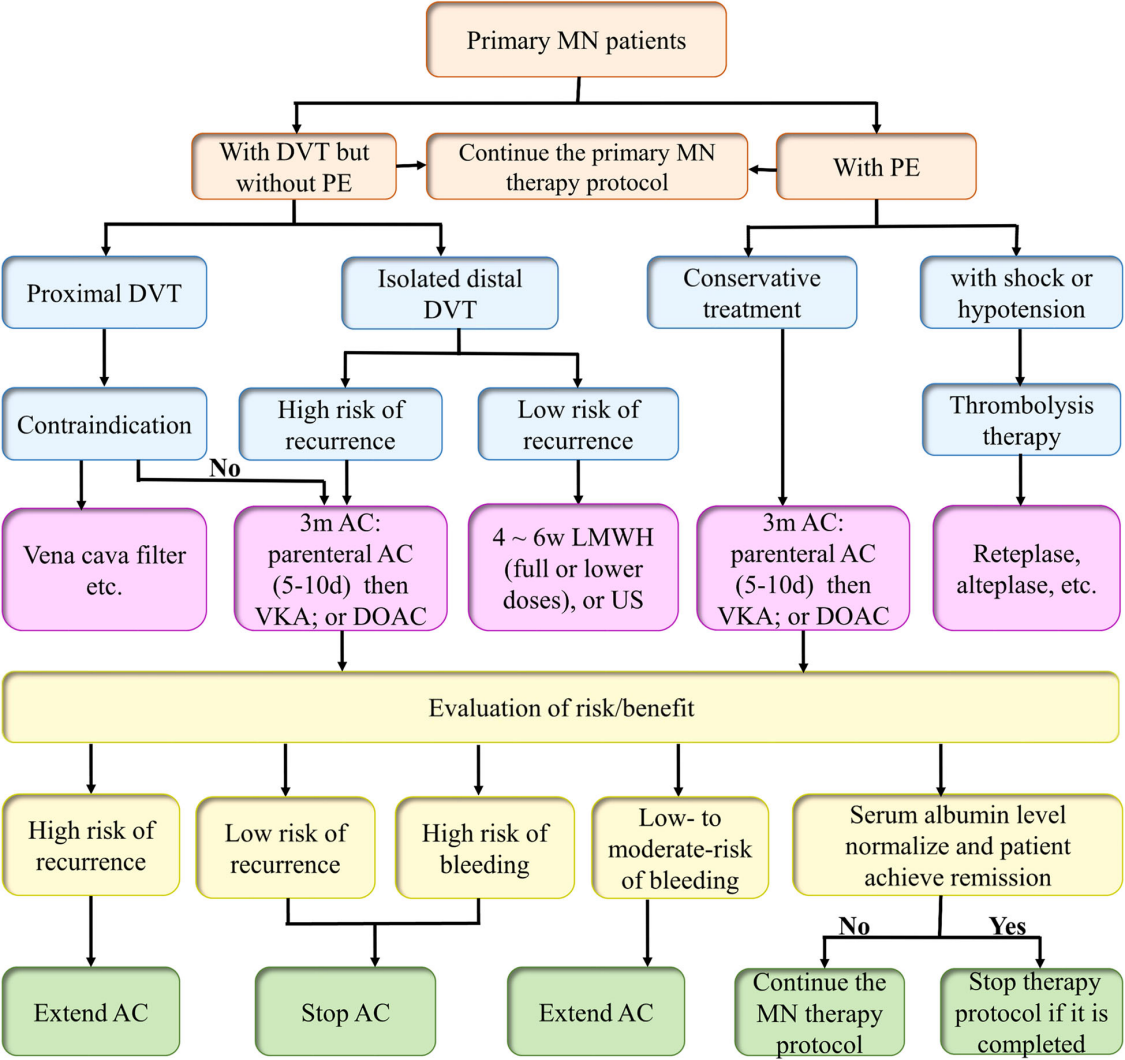
Proposed algorithm to guide the decision on anticoagulant management in primary MN patients with VTE. MN: membranous nephropathy; VTE: venous thromboembolic event; DVT: deep venous thrombosis; PE: pulmonary embolism; AC: anticoagulation; m: month; d: days; VKA: vitamin K antagonists; DOAC: direct oral anticoagulant; w: weeks; LMWH: low molecular weight heparin; US: ultrasound surveillance

Based on the VTE-related guidelines and some literature, we found that the treatment of VTEs (not provoked by surgery or cancer) in patients with primary MN is almost similar to that in the general population with VTEs. Of note, D-dimer levels may be affected by proteinuria and may not be an independent predictor of stopping anticoagulant therapy in patients with primary MN with VTEs. Moreover, the reduction of proteinuria and the increase of serum albumin are also important goals for the treatment of primary MN with VTEs [37–39]. It is necessary to continue the previous steroid therapy or to combine it with immunosuppressive agents over the entire treatment period until the primary MN treatment protocol is completed. However, there is currently no clinical trial data on how long anticoagulation should last in this patient population. In our own opinion, for primary MN patients with VTEs, one potential approach is to receive anticoagulation at least 3 ~ 6 months (if there are no contraindications) until serum albumin levels normalize and patients achieve remission. If there is a strong indication for anticoagulation therapy, such as atrial fibrillation (AF) or multiple VTEs, indefinite anticoagulation therapy is recommended (unless there is a contraindication). Of course, we believe that a comprehensive risk factor assessment is essential, and any anticoagulation therapy needs to be continuously monitored during the entire treatment period. Figure 2 shows the anticoagulant management algorithm in primary MN patients diagnosed with a VTE.

Apixaban, as a DOAC, provides an effective and safe regimen for the initial and long-term treatment of DVT and PE compared to conventional therapy (subcutaneous enoxaparin, followed by warfarin) and reduces the risk of recurrent DVT and PE compared to placebo following initial therapy [40, 41]. A retrospective cohort study and the seminal ARISTOTLE trial showed that among the patients with ESRD and AF on dialysis or those with only AF, apixaban use may be associated with a lower risk of stroke, bleeding and death compared with warfarin [42, 43]. Furthermore, a meta-analysis showed that major bleeding and fatal bleeding were significantly lower in DOAC-treated patients [44]. DOACs were at least as effective as and possibly safer than parenteral drug/VKA therapy.

Notably, apixaban and rivaroxaban are highly bound to plasma proteins (87, 90% ~ 95%) in humans, and serum albumin is the main binding component. If these drugs are considered for patients with hypoalbuminemia, the protein binding rate is an important consideration in primary MN [45, 46]. At present, although no pharmacokinetic study was available and no clinical trial had been performed in primary MN, some case reports reported the successful use of DOACs for the treatment of clinically evident thrombosis or thromboprophylaxis in primary MN [46]. Although the literature on DOAC use in NS is limited, the preliminary experience seems promising. Therefore, we believe that DOACs may be considered for anticoagulant therapy in primary MN with VTEs. Notably, the choice of DOACs ought to be individualized based on specific patient factors, for instance, renal function, and the risk of thrombosis and bleeding.

Since most of the DOACs are largely excreted through the kidneys, the degree of renal impairment is an important factor in determining whether DOACs can be used and in selecting the appropriate doses. Severe renal impairment in primary MN patients [creatinine clearance (CrCl) < 15 ml/min] is a contraindication for DOACs. Mild renal impairment (CrCl > 50 ml/min) has little effect on DOAC pharmacokinetics. If CrCl is between 15 and 50 ml/min, discretionary reduction is required [47]. Since limited data are available, it is necessary to conduct risk stratification and careful follow-up of these patients to ensure a net clinical benefit of thromboprophylaxis. It is worth noting that DOACs are not suitable for all individuals with severe kidney disease [48].

### Primary MN with complications of ACS

The hypercoagulability associated with NS can result in the development of an occlusive coronary artery thrombus in absence of atherosclerotic coronary artery disease [49]. ACS, ST-elevation myocardial infarction and non-ST-elevation myocardial infarction is not a very rare complication; sometimes, it can be the first manifestation in primary MN [49–52]. the accurate diagnosis of the complication is imperative for suitable management and secondary prevention in primary MN [53]. Currently, neither primary MN-related nor ACS-related guidelines can provide the recommendations associated with the management of anticoagulant and antiplatelet agents in primary MN patients with the complication of ACS. The optimal management of the medications is still under discussion.

According to the guidelines, once ACS is diagnosed, the bleeding risk in addition to the ischemic risk should be carefully evaluated during the acute phase of the ACS. It is recommended that the earliest possible drug administration is dual antiplatelet therapy in the prehospital setting. Furthermore, ACEIs/ARBs, statins and β-blockers should be administered as soon as possible after admission [54–56]. No matter which type of therapy is applied (conservative treatment, reperfusion therapy with percutaneous coronary intervention or thrombolytic agents, etc.), it is usually recommended to use UFH or enoxaparin or bivalirudin immediately after conservative treatment or reperfusion therapy [57, 58]. The details are as follows: in ST-elevation myocardial infarction patients who receive thrombolytic therapy, UFH or enoxaparin should be used around the time of reteplase or alteplase therapy. For patients with conservative treatment or percutaneous coronary intervention, bivalirudin is the better choice than UFH if patients are at a high risk of bleeding [55]. After hospital discharge, ACEIs/ARBs, statins, β-blockers and low doses of aspirin should be indefinitely continued, and 12 months of P2Y12 receptor antagonist therapy is needed. Stopping P2Y12 receptor antagonist therapy can be considered in patients with a high risk of bleeding after 6 months. The statins should not be stopped blindly and the dosage should not be reduced even when low-density lipoprotein cholesterol is lower than 2.07 mmol/L.

**Fig. 3:**
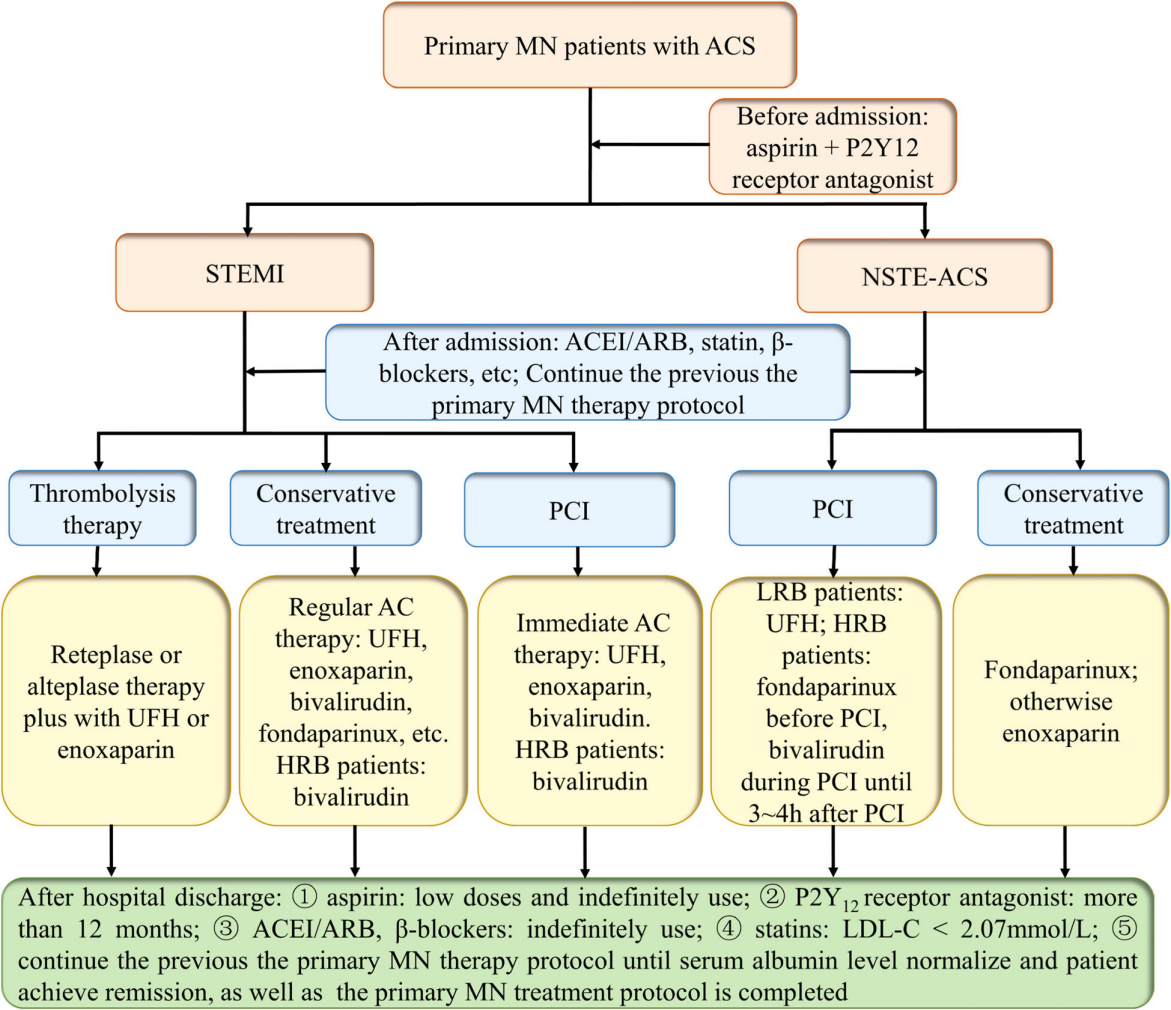
Proposed decision-making algorithm of medication in primary MN patients with ACS. MN: membranous nephropathy; ACS: acute coronary syndrome; STEMI: ST-elevation myocardial infarction; NSTE: non ST-elevation; ACEI: angiotensin-converting enzyme inhibitor; ARB: angiotensin receptor blocker; PCI: percutaneous coronary intervention; AC: anticoagulation; UFH: unfractionated heparin; LRB: low risk of bleeding; HRB: high risk of bleeding; h: hours; LDL-C: low-density lipoprotein cholesterol

We analyzed the existing case reports and reviews that detailed the treatment process and found that, in general, in primary MN patients with a confirmed diagnosis of ACS, the treatment regimen of ACS was almost similar to that of the general population with ACS. It was noteworthy that a majority of primary MN patients with ACS continued the previous primary MN treatment protocol during the entire treatment period until they obtained remission (partial or complete remission) and the protocol was completed [49–52]. Provided that there is an additional strong indication that requires anticoagulant therapy, over and above that of their primary MN with ACS, the patient might have extra lifelong anticoagulation therapy [49]. Since we were reviewing the treatment options in the existing case reports and reviews, the views we presented might have limitations. The treatment regimen of primary MN patients with ACS should be individualized based on specific patient factors. The medication management of primary MN patients with ACS is shown in Fig. 3.

### Primary MN with complications of IS

IS is one of the most severe complications of NS. The hypercoagulable state may be the main contributing factor of IS in NS [59]. A cohort study showed that IS accounted for 45% of the ATEs in Chinese patients with primary MN [7]. IS usually occurs at the initial onset or upon the relapse of primary MN and it lacks specific clinical manifestations. Therefore, early diagnosis and intensive treatment are crucial to the prognosis of IS in primary MN [60].

According to the related recommendations, the therapeutic strategies for IS in the general population include endovascular intervention, thrombolysis, antiplatelet therapy and anticoagulation therapy [61]. After the assessment of risks and benefits by the physician, heparin or VKA therapy can be considered in patients with a hypercoagulable state 24 h after thrombolysis or endovascular intervention. The discontinuation of the drugs can be considered until the INR is in the range of 2.0 ~ 3.0. It is usually unnecessary for patients who are not in a hypercoagulable state to receive early anticoagulation therapy. Recommendations suggest starting aspirin therapy 24 h after thrombolysis. One to 3 months of dual antiplatelet therapy is recommended for patients after endovascular intervention. For patients who are prepared to receive conservative treatment, it is generally not recommended to undergo anticoagulation therapy; they should begin aspirin therapy as soon as possible. If patients suffer from a slight IS (course of the disease is within 24 h and NIHSS score is ≤3), continuing dual antiplatelet therapy for 21 days may be an optimal choice. In addition, it is recommended that, for all IS patients, the indefinite use of aspirin or clopidogrel is an acceptable strategy for the secondary prevention of IS [62, 63].

**Fig. 4:**
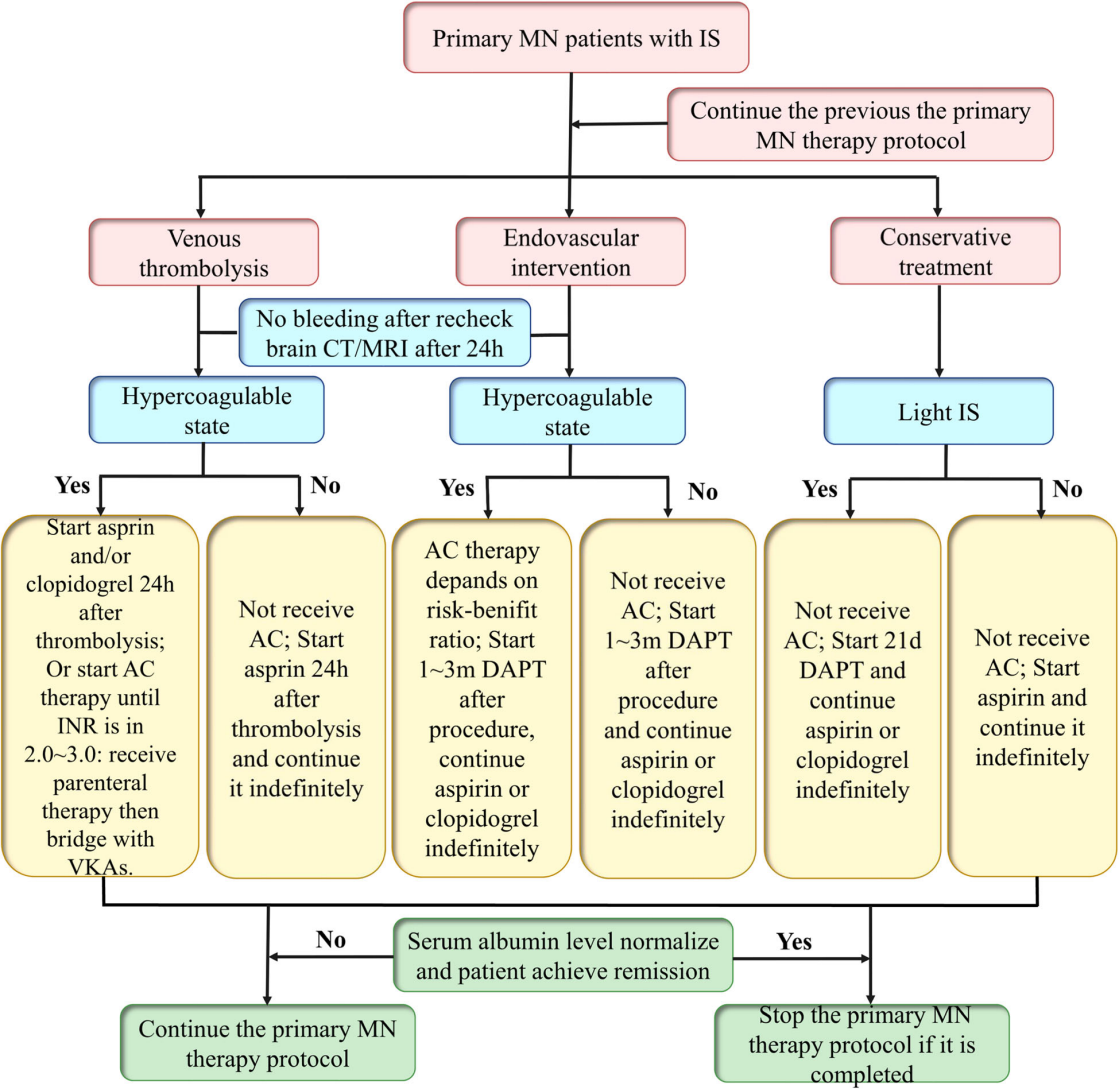
Authors’ suggestions for medication management in primary MN patients with ischemic stroke. MN: membranous nephropathy; IS: ischemic stroke; AC: anticoagulation; h: hours; m: month; INR: international normalized ratio; UFH: unfractionated heparin; LMWH: low molecular weight heparin; DAPT: dual antiplatelet therapy

However, the evidence for the efficacy and safety of medication management in primary MN with IS has yet to be determined. We found that in the existing case reports and reviews, once the diagnosis of IS was established in primary NM, the treatment for IS was largely similar to the therapy for IS in the general population. Simultaneously, patients continued the previous primary MN treatment protocol until they achieved remission and the protocol was completed (Fig. 4) [6, 59, 64, 65].

According to individuals’ conditions, some patients are more inclined to receive anticoagulant therapy (parenteral therapy and then bridge with VKAs) after the thrombolysis therapy or the endovascular intervention [6, 65]. Since platelet hyperaggregability plays an important role, antiplatelet therapy may be useful in reducing the risks of thromboembolic disease in primary MN. Some patients are treated with dual antiplatelet agents auch as aspirin and clopidogrel after IS-related supportive care [59, 64]. To what extent the use of anticoagulants and antiplatelet agents can prevent IS in primary MN is yet unknown. Further numerous clinical data are needed to determine the role of anticoagulants and antiplatelet agents in the primary and secondary prevention of IS in primary MN.

## Discussion

### Summary of evidence

In this review, we eventually identified 65 primary studies addressing prophylactic and therapeutic anticoagulation therapy in primary MN. Our findings revealed a paucity of research focusing specifically on prophylactic or therapeutic anticoagulation in primary MN patients with a high risk of thrombosis or with thromboembolic complication and a limited number of clinical trials in this area. We found that personalized prophylactic aspirin or warfarin could be considered to prevent ATEs and VTEs in primary MN patientsvwith serum albumin < 3.2 g/dl. Moreover, the treatment regimen of thromboembolic complications in primary MN patients was similar to that in the general population with thromboembolic events. Since more evidence indicated that DOACs provided an effective and safe regimen for anticoagulant treatment in NS compared to conventional therapy, they might be a promising anticoagulant in primary MN. Last but not least, the patients should continue the previous primary MN treatment protocol during the entire treatment period until they achieve remission, the protocol is completed and the underlying diseases have resolved.

### Limitations

There were some limitations in the present review. Most of the evidence for prophylactic anticoagulation recommendations for primary MN was derived from retrospective studies. Randomized controlled trials should be conducted to support this evidence. The views on primary MN patients with ACS or IS presented in the review were based on the existing reviews, case reports, ACS- and IS-related guidelines, which lacked convincing direct evidence. The current evidence supporting prophylactic and therapeutic anticoagulation is too weak to better meet the clinical needs in primary MN patients. The optimal, standardized approach has not been clearly established. The issues discussed in the present review might be valuable as a basis for a discussion leading to a consensus statement by scientific societies or for an update of guidelines on the management of IMN. In other words, it is necessary to perform multicentered randomized controlled trials to support the present review.

## Conclusion

The utility of prophylactic aspirin or warfarin may have clinical benefits for the primary prevention of thromboembolic events in primary MN patients with hypoalbuminemia.

## Data Availability

Not applicable.

## Abbreviations

ACEI/ARB: angiotensin converting enzyme inhibitor/angiotensin receptor blocker
ACS: acute coronary syndrome
AF: atrial fibrillation
ATE: arterial thromboembolic event
ATRIA: anticoagulation and risk factors in atrial fibrillation
CrCl: creatinine clearance
CVE: cardiovascular event
DOAC: direct oral anticoagulant
DVT: deep venous thrombosis
ESRD: end stage renal disease
HAS-BLED: hypertension, abnormal renal/liver function, stroke, bleeding history or predisposition, labile international normalized ratio, elderly, drugs/alcohol
INR: international normalized ratio
IS: ischemic stroke
LMWH: low molecular weight heparin
MN: membranous nephropathy
NS: nephrotic syndrome
PE: pulmonary embolism
py: patient-years
RVT: renal vein thrombosis
UFH: unfractionated heparin
VKA: vitamin K antagonists
VTE: venous thromboembolic event
